# NurseNet: Monitoring Elderly Levels of Activity with a Piezoelectric Floor

**DOI:** 10.3390/s19183851

**Published:** 2019-09-06

**Authors:** Ludovic Minvielle, Julien Audiffren

**Affiliations:** Centre de mathématiques et de leurs applications, CNRS, ENS Paris-Saclay, Université Paris-Saclay, 94230 Cachan, France

**Keywords:** deep learning, piezoelectric sensor, floor sensor, pressure sensor, activity monitoring, sparse dictionary learning

## Abstract

Monitoring the activity of elderly individuals in nursing homes is key, as it has been shown that physical activity leads to significant health improvement. In this work, we introduce NurseNet, a system that combines an unobtrusive, affordable, and robust piezoelectric floor sensor with a convolutional neural network algorithm, which aims at measuring elderly physical activity. Our algorithm is trained using signal embedding based on atoms of a pre-learned dictionary and focuses the network’s attention on step-related signals. We show that NurseNet is able to avoid the main limitation of floor sensors by recognizing relevant signals (i.e., signals produced by patients) and ignoring events related to the medical staff, offering a new tool to monitor elderly activity in nursing homes efficiently.

## 1. Introduction

### 1.1. General Introduction

In the recent years, the notion of frailty has received increased attention from the medical community as an early sign of significant health deterioration in the elderly population [1,2]. The authors of [1] defined frailty as a clinical syndrome in which three or more criteria are present among unintentional weight loss, self-reported exhaustion, weak grip strength, slow walking speed, and low physical activity. The early detection of this syndrome is considered important by the medical community for prevention purposes, and targeted therapy strategies have been shown to reduce the consequences of frailty significantly [3].

Nursing home residents are among the most vulnerable when it comes to frailty [4]. Indeed, people living in elderly care facilities are more likely to have disabilities than other elderly people. They also tend to be more isolated from the rest of the population and do less physical activity than the general elderly population [5]. The lack of exercise is particularly damaging, as it has been shown that physical activity reduces muscle weakness, increases mobility, improves neuronal health, limits frailty, and reduces overall risk of death [6,7]. Even short daily practice such as thirty minutes of walking is linked with significant improvement [8]. Consequently, the monitoring physical activity of the elderly in nursing homes is key, particularly as it allows focusing resources on people who are particularly vulnerable to frailty.

Recently, there has been significant interest in the development of smart systems to monitor individuals in their daily life [9]. Important aspects of such systems include the intrusiveness, the reliability, and the ability to perform different tasks. Here, intrusiveness is defined as both the capacity to be hidden from the patient and the respect of her/his privacy [10]. Some sensors relying on technologies such as a camera [11] or microphones [12] have been shown to achieve great performance in their respective task (counting people, detecting events, etc.) at the cost of serious privacy issues. Others have used accelerometers, which are less intrusive and provide reliable signals [13,14], but wearable technology is (A) constrained by individual willingness and (B) may be rendered ineffective if individuals forget to wear them [15]. Floor sensors on the other hand resolve these issues by being unobtrusive while still providing exploitable signals. However, the main drawback of floor sensors is their *a priori* inability to distinguish between individuals. This is particularly problematic in nursing homes as most of the activity is generally generated by medical staff.

To solve this problem, in this paper, we introduce our system named the Non-invasive Unit Recognition System for the Elderly (NurseNet), which combines a piezoelectric floor sensor and a deep learning-based algorithm to recognize non-elderly activity with very high accuracy (AUC 0.94). This paper is organized as follows. Section 2 first gives the settings of our installation in the nursing home and introduces our algorithm and its training procedure. The performance of NurseNet is evaluated in Section 3 by computing its accuracy over a database containing multiple types of activity. Finally, the main advantages and drawbacks of our system are discussed in Section 4.

### 1.2. Related Work

#### 1.2.1. Gait Sensing

The quantification of gait is hardly new [16,17,18], and a large variety of sensors has been used to record walks. Systems can be classified between wearable sensors and non-wearable, the latter being divided between image-based systems and other systems. The most popular are wearable sensors, as evidenced by the large literature on the subject [18,19]. These systems are mostly based on accelerometers, which dominate the field, but also gyroscopes, foot switches, pressure soles, or combinations of these. Accelerometers are indeed inexpensive sensors that give reliable signals and are more scalable than foot switches or soles [16].

Concerning non-wearable sensors, video-based systems seem to give good results when analyzing gait. However, considering the high intrusiveness of these solutions, they are better suited for clinical applications rather than daily monitoring. Recent works proposed systems based on WiFi sensing [20,21]. Based on the measurement of the WiFi signal after reflection on objects, many applications were proposed, but this technology seems to be in its early stages and suffers from issues such as the difficult availability of the needed metric to perform the sensing and external interferences [21]. When it comes to floor technologies, various technologies have been used. Vibration-sensor systems are very easy to install, and previous works have shown that they can be used to separate individuals with high accuracy [22,23]. However, the signal is subject to variation according to the device’s location and the size of the sensing area, which may require significant additional training for each new installation. Floor-covering solutions [10], although requiring more effort in their deployment, have the advantage of being consistent since the sensing is equivalent at any location. These systems are mainly made of binary switch sensors [24,25], capacitive sensors [26,27,28], and pressure sensors, the latter being either piezoresistive (the material resistance is modified when put under pressure) [29] or piezoelectric (the material emits a charge or a voltage when stressed) [30,31]. When implemented, these systems have different resolutions, from only one sensor to a grid of 1 cm × 1 cm tiles. However, high resolution leads to implementation issues and higher costs. Depending on the sensor and resolution, the emitted signals can provide sufficient information for complex tasks, but can also suffer from external perturbations [10]. Our system is based on a thin piezoelectric polymer that acts as a pressure sensor and comes in bands. It is unobtrusive and gives a reliable signal while being relatively easy to install, all at a reduced cost due to precisely the ease of installation, but also the availability of its components (processing unit, sensors, and flooring) for large-scale installations and the fact that the system comes as an all-in-one solution.

#### 1.2.2. Gait Analysis

The main families of analyses developed in previous works include: (A) the use of biomechanical features, such as single/double stance time [32]; (B) the use of descriptors derived from mathematical models [33]; and more recently, (C) the use of machine learning-based algorithms that perform end-to-end learning on gait signals [34]. Several walk detection systems rely on gait decomposition into several phases, trying to detect two to eight phases [16] depending on the sensor and the precision level needed for their application. These methods can be based on heuristic rules (e.g., thresholds [35]), signal processing techniques (e.g., Pan–Tompkins peak detection [36]), or probabilistic approaches (e.g., HMM-based algorithms [37]). However, most methods are designed to characterize gait in a controlled environment where no challenging type of signal can infer (i.e., signal that may seem like what is to be detected). Hence, when dealing with daily activities, walk detection becomes a harder task [38,39]. Another segment of step detection aims at recognizing a step signal as a whole (i.e., without considering any phase in it) in order to perform walk detection within more challenging environments or to use walk signals for patient monitoring. These methods are generally based on machine learning techniques and seem more suited to such tasks [40,41], but to the best of our knowledge, no previous work has classified activities using a floor piezoelectric sensor combined with machine learning techniques.

#### 1.2.3. Sparse Coding and Dictionary Learning

In recent years, dictionary learning and sparse coding techniques have been successfully applied in a wide range of topics, including image classification [42,43], image restoration [44], and signal processing [45], in particular for one-dimensional signal analysis [46]. The main idea behind these representations is to learn a dictionary conjointly containing the patterns observed in the signal and sparse activations that encode the temporal or spatial locations where these patterns occur. Previous works have applied this method to walk-related problems: Poschadel et al. [47] used the accelerometer signal reconstructed from sparse coding to perform gait classification between healthy and movement impaired individuals; Zhang et al. [40] also aimed at gait classification by applying D-KSVD techniques to produce a dictionary that is both representative and discriminative. Following the same idea, we use convolutional dictionary learning to build the first layer of NurseNet in order to construct a relevant representation.

#### 1.2.4. Signal Processing with Deep Learning

Convolutional Neural Networks (CNN) have been successfully used to detect objects in images [48,49,50,51,52], hence superseding previously existing techniques [53]. Among them, recent networks such as YOLO [52] and Faster R-CNN [51] have been shown to find and classify multiple objects with high accuracy in images with a small time cost. However, significantly less attention has been given to the application of CNN on one-dimensional signals, despite such signals being widely represented in medical recordings [54]. Previous works generally transformed one-dimensional signals into an image using spectrograms [55] or directly used CNN without transforming the data [56]. To the authors’ knowledge, this is the first work to combine dictionary learning, transfer learning, and CNN to classify activities using the floor sensor as the sole input. As shown in Section 2.2, the combination of these three steps is revealed to be necessary due to the highly-irregular nature of the signal.

## 2. Materials and Methods

### 2.1. NurseNet Hardware

In this subsection, we present the floor sensor that was used in NurseNet. The piezoelectric principle (on which the device was based) is outlined, then the unit is described in more depth.

#### 2.1.1. The Piezoelectric Principle

The piezoelectric principle, when simplified, can be explained by the relation $d=\frac{Q}{F}$, with *d* being the piezoelectric coefficient, *Q* the charge, and *F* the force received by the material. A piezoelectric material emits charges when stressed (or squeezed) and on the contrary can shrink (or expand) when submitted to a electric field. Such a device can fit multiple purposes such as speakers or motors when using its deformation under a electric field, to microphones and sensors when using the electric charge emitted when submitted to a force. Our system was based on the latter use, and the piezoelectric material acted as a pressure sensor. One significant advantage of this technology is that it does not need a power supply to generate a signal.

#### 2.1.2. The NurseNet Unit

**Figure 1:**
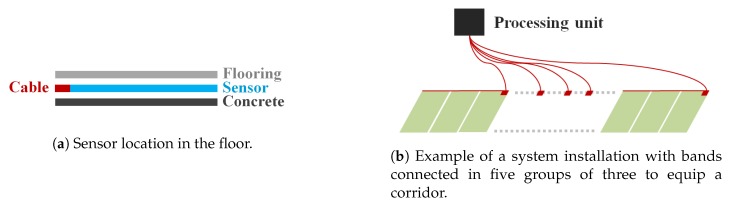
Pressure sensor installed in the floor.

The sensor itself was a thin (about 1 mmr) polymer that comes in bands of 60 cm wide and placed directly under the flooring (see Figure 1a). The bands were initially rolls of about 100 m that could be cut every 30 cm. Once cut, bands can be connected together without limitation in their number before the signal was recovered by the processing unit (see Figure 1b). In NurseNet, the processing unit had 8 entry channels, allowing each unit to process a large surface by connecting bands. This allows great flexibility of the system to any scale while keeping a certain ease of installation.

The processing unit was designed to process the signal from end to end (from basic filtering to event detection) and to communicate with servers (sending signal recordings) and the nursing home (sending information such as activity reports or alarms). When conveyed to the unit, the signal first passed through an analog charge amplifier and was converted to numerical values before further processing. The unit was equipped with a 32-bit 500-MHz processor, accompanied with 256 MB of RAM, and 500 MB of local storage.

It should be noted that compared to the theoretical piezoelectric principle law, the sensor presented some variability in the piezoelectric coefficient *d*. Indeed, although *d* seemed to remain constant when put under humidity or high temperature (less than 40° Celsius), it could vary depending on where the impact was located on the floor. A difference of 9% between the max and min value of *d* was observed in controlled experiments [57]. Besides, when implemented, the system can suffer perturbations due to the way sensors, electrodes, and upper layers were installed. These elements that come with the implementation in real conditions can result in alterations to the resulting signals when compared to similar devices used in laboratory conditions [10].

### 2.2. The NurseNet Algorithm

In this subsection, we present our neural network NurseNet, which is used to identify and characterize activity signals, particularly between medical staff and elderly individuals. This subsection is organized as follows. First, the structure of NurseNet is introduced. Then, the data embedding used to encode the signals is described, and finally, the subnetwork used as the first layers of NurseNet is explained. Most of the strategies developed in the training process aim at circumventing our main issues, which are the small Signal-to-Noise Ratio (SNR) (approximately 20 dB in our walking dataset) and the external alterations of the signal.

#### 2.2.1. General Classifier

**Figure 2:**
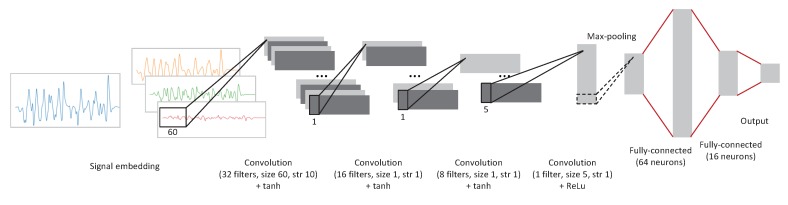
Architecture of the overall CNN.

This subsection details the architecture of NurseNet and its inner workings. The main idea of NurseNet is that regardless of the type of activity recorded on the floor sensor, it is very likely to be mostly made of walks. This is why, as discussed later in this section, a significant part of the training process aimed at training the network to recognize gait and in particular the gait of the medical staff. NurseNet is a CNN whose structure is presented in Figure 2. It is a sequential network made of:

- One 1D convolutional layer with 32 filters of size 60, with a stride of 10, followed by a BatchNorm layer, with the activation function hyperbolic tangent (tanh).
- One 1D convolutional layer with 16 filters of size 1, with a stride of 1, followed by a BatchNorm layer, with the activation function tanh.
- One 1D convolutional layer with 8 filters of size 1, with a stride of 1, followed by a BatchNorm layer, with the activation function tanh.
- One 1D convolutional layer with 1 filter of size 5, with a stride of 1, with the activation function Rectified Linear Unit (ReLU) [58]. It was followed by a Maxpool layer of size 5.
- One fully-connected layer, with an output of dimension 64, with activation function ReLU.
- One fully-connected layer, with an output of dimension 16, with activation function ReLU.
- One fully-connected layer, with an output of dimension 1, with activation function sigmoid.

The output of the network is the probability for a signal to be generated from elderly activity. Note that the input of NurseNet is not the raw signal, but instead a transformation of it (see Section 2.2.2).

**Sub-networks.**NurseNet can be seen as the combination of the following two sub-networks. The first one, named the Step Proposal Network (SPN), is made of the first three convolutional/BatchNorm layers. SPN is a full CNN inspired by the region proposal network, which is the first part of the Faster R-CNN algorithm [51]. At the end of this sub-network, the output is constituted of features computed on each window of the signal embedding. The second part of the network is made of a convolutional/Maxpool pair of layers, followed by three classical fully-connected layers. The role of this last part of the network is to use the previously extracted features to classify signals as *staff* or *elderly*.

**Training.** As mentioned before, the training of NurseNet was rather complex and involved multiple stages. This was due to the small signal-to-noise ratio, the perturbations discussed in the previous section, and the limited size of the dataset. The first phase of the training was to learn the embedding of the data. This is detailed in Section 2.2.2. Then, SPN was trained separately on a different task, in order to take advantage of the gait structure (see Section 2.2.3). The resulting weights were used to initialize the first three layers of NurseNet, and these layers were frozen during the rest of the training process to limit the number of free parameters, henceforth reducing overfitting [59]. Finally, the rest of the network was initialized using i.i.d. centered Gaussian variables with a standard deviation of 0.2. For the training phase, regularization was done using dropout [60] (with p=0.5) after each fully-connected layer, except the last one. Given $X_{i}$, the embedding of each signal $\hat{y_{i}}$, the image of $X_{i}$ through the network, and $y_{i}$ the corresponding labels (y=0 if and only if $X_{i}$ is an elderly activity), the loss function L is the classical binary cross entropy:

(1) $$L=\sum_{i}1_{y_{i}=1}\log\left(\hat{y_{i}}\right)+1_{y_{i}=0}\log\left(1-\hat{y_{i}}\right).$$

The weights of NurseNet were optimized using backpropagation and stochastic gradient descent with a learning rate of $10^{-5}$, decreasing geometrically (×0.9) every 10 epochs, and the momentum strategy proposed by Nesterov [61]. The learning was stopped using the early stopping principle [62].

#### 2.2.2. Data Embedding

The first step of our classification algorithm was the data embedding, which consisted successively of preprocessing the data and encoding the resulting signal using convolution with atoms of a pre-learned dictionary. The preprocessing phase was made of a classical tool that aimed at denoising and cleaning the data. However, our approach of data embedding is rather new: while we used convolutional sparse coding to learn the custom dictionary, a standard approach since sparsity has been shown to produce more relevant atoms (see [63] and the references therein), the actual embedding of the data was done with (non-sparse) convolution.

The reasoning behind this approach is as follows. It has been shown that the first layers of a CNN tend to learn general feature extractors: for example, in image processing, the first layers usually exhibit features similar to Gabor filters and color blobs [64]. The aim of our data embedding phase was to replace the first layers of the CNN with some already trained filters, here the atoms of the dictionary. Since the training was done on a small dataset, reducing the number of layers in the network may improve the results. Hence, in order to mimic the behavior of the first layers of the CNN, regular convolution was used instead of convolutional sparse coding. All data embedding details were given below.

**Preprocessing.** It has been shown that preprocessing improves the performance of CNN networks [65], particularly when dealing with a small labeled dataset. The preprocessing of data was done as follows. Let ${\left(C_{t}^{k}\right)}_{t=1}^{T}$ denote the signal produced by the channel *k* (1≤k≤K=5), where t=1,…,T represent the data points recorded with a frequency of 100 Hz.

1. Each channel ${\left(C_{t}^{k}\right)}_{t=1}^{T}$ was filtered with a low-pass Butterworth filter with a 10-Hz cutoff frequency, fifth order, and zero lag. This step aimed to limit the amount of electronic noise present in the signal, as the 10-Hz cutoff frequency is the reference in gait-related signals [66].
2. The linear trend of each channel was removed using a least squares model.
3. Each channel whose signal maximum amplitude was small was then set to zero, as the channel was assumed to only account for noise.
4. The resulting signal s was obtained as the sum of all the channels:
  $$s≐{\left(C_{t}\right)}_{t}={\left(\sum_{k=1}^{K}C_{t}^{k}\right)}_{t}$$

**Figure 3:**
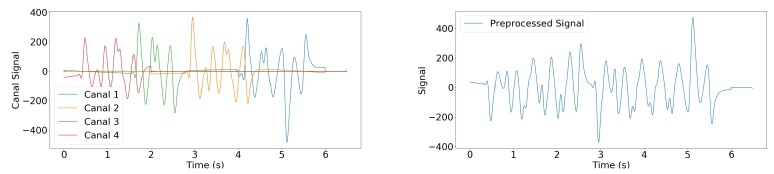
Example of raw (**left**) and preprocessed (**right**) signals resulting from medical staff walking on the sensor. In this case, no channel was set to zero during Step 3.

Figure 3 shows an example of the resulting signal.

**Signal embedding.** Data augmentation is a key part of training complex neural networks, particularly when only small labeled datasets are available [67]. However, these techniques are difficult to apply in our setting, as it is unclear that commonly-used transformations would preserve the nature and structure of the data. For instance, a re-scaled step signal may be confused with a fall signal, as the amplitude of the applied force is the main difference between the two signals (see [68]). In our case, we are dealing with a small dataset, which is composed of complex signals, thus making end-to-end learning a difficult task. To circumvent this difficulty, the data were first transformed using convolution with atoms learned from Convolutional Dictionary Learning (CDL) in order to extract key elements and features of the signal. This first phase was intended to improve the quality of the CNN input to balance out the limited amount of available data.

**Convolutional dictionary learning.** CDL is defined as the learning of a set of atoms to best represent data (e.g., images or one-dimensional signals) while insuring a sparse representation. Given a vector s that contains data to be represented, the objective is to find atoms $d_{m}$ and activation signals $x_{m}$ such that the reconstruction is accurate, i.e., $s\approx \sum_{m}x_{m}*d_{m}$. Solving CDL generally uses the following objective function, referred to as convolutional basis pursuit denoising [46]:

(2) $$\underset{x_{m},d_{m}}{arg min}\frac{1}{2}∥\sum_{m}x_{m}*d_{m}{-s∥}_{2}^{2}+\lambda \sum_{m}{∥x_{m}∥}_{1}$$

Previous works have shown that many walk alterations can be detected and quantified by studying the characteristics and variety of steps [69]. Following this idea, CDL has been successfully used to quantify and evaluate gait [47]. However, it is worth noting that CDL is generally used on signals derived from accelerometers, which have been proven to be a robust and reliable way to record walks. In our setting, the recording was of lower quality, and nearly-identical steps, such as the ones produced by the walking of young healthy individual [70], were recorded with significant variations between them (see Figure 3). Besides, a large number of signals in the dataset contained elements that were not related to steps (e.g., pushing a wheelchair or a cart). Finding a suitable dictionary was therefore significantly more challenging, and this is why atoms were created by selecting signals that were almost entirely walk related.

**Learning step atoms.** As previously said, in order to learn atoms relevant to our step description process, we used a CDL approach on a subset of the walk signals, to guide the learning towards step-related atoms. Equation (2) shows that when learning a dictionary, we learn at the same time the dictionary and sparse representations of input signals. The standard procedure of dictionary learning is to alternate between a sparse coding step (i.e., updating the sparse representation of the data according to the current dictionary) and a dictionary update (i.e., updating the dictionary according to the current sparse representation). This means that (2) is solved successively with $d_{m}$ fixed (sparse coding) or $x_{m}$ fixed (dictionary update). We used the method of Garcia-Cardona and Wohlberg [71], which is derived from the Alternating Direction Method of Multipliers (ADMM). Proposed by Boyd et al. [72], ADMM solves problems of the form:

(3) $$\underset{x,y}{arg min}f\left(x\right)+g\left(y\right)\;\mathrm{such}\;\mathrm{that}\;Ax+By=c$$

**Figure 4:**
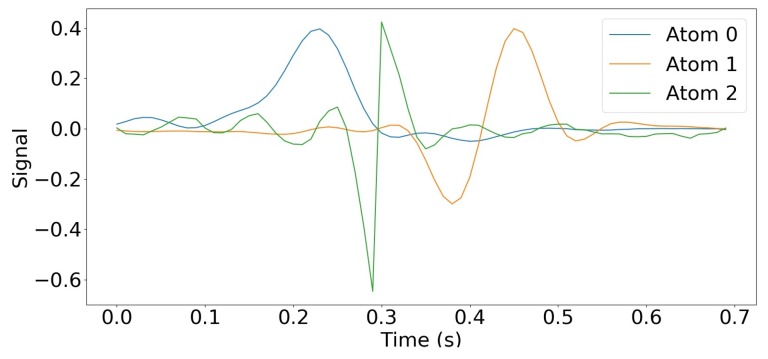
Dictionary learned using Equation (2). The amplitude of the atoms is small compared to the signal because they are normalized.

By using the augmented Lagrangian multipliers. Adapted to sparse coding and dictionary update with a specific coupling between the two steps (named consensus), this method proved to be efficient among the existing ones [46]. We used the implementation of Wohlberg [73] of the algorithm, available in a Python package named SPORCO (SParse Optimization Research COde). The number of atoms *m* was considered a hyperparameter of our CDL problem, and we chose it using cross-validation. Our experiments showed that m=3 achieved the best trade-off between the quality of the reconstruction of the training set and the one of the validation set, while maintaining the sparsity of the representation. Using less atoms led to either poor reconstruction or non-sparse representation of the training set, whereas using more atoms significantly decreased the reconstruction quality of the validation set. These three atoms were 0.7 s long, which corresponded to an upper bound of the duration of a step in the walking of a healthy young individual [74]. They were obtained with λ=5 and 2000 iterations during the optimization process. Figure 4 shows the resulting dictionary.

**Convolutional dictionary embedding.** As discussed above, while we used a sparsity constraint on the dictionary learning process, the actual embedding of the input data s into $S^{d}$ was done with standard convolution:

(4) $$S^{d}≐{\left(s*d_{m}\right)}_{1\leq m\leq 3}$$

**Figure 5:**
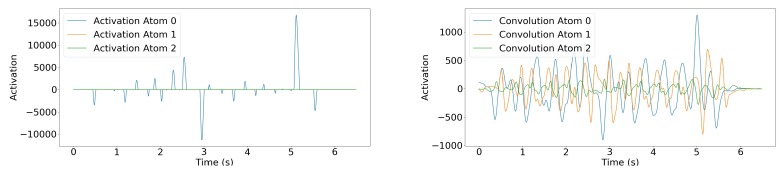
(**Left**) Convolutional sparse decomposition of the signal of Figure 3. Note that for this individual, only Atom 0 is used. (**Right**) Convolution of the same signal with each atom. For this signal, only Atom 0 was used in the sparse coding solution, but this differs in other signals. Moreover, it is interesting to note that since Atom 0 is mildly similar to a Gaussian kernel, the result of the convolution of the signal with Atom 0 is close to a smoothed version of the original signal.

Resulting in a three-channel encoding of the signal. Figure 5 shows an example of the data encoding process.

#### 2.2.3. Pre Training Weights with Step Detection

This subsection discusses the idea behind the first sub-network of NurseNet and presents its dedicated training process.

As discussed before, this sub-network is inspired by the Region Proposal Network (RPN). The idea behind RPN is to help the main network of Faster R-CNN [51] focus only on interesting parts of an image. To this end, a sliding window was passed on a feature map of the input image. On this sliding window, boxes of different scales and ratios (in total *K*) were used as region proposals, and these regions were evaluated through two layers. The first one output the probability of a box to be an object and the other, one gave corrected coordinates of the box. This method allowed reducing the number of false examples that may mislead the training process and greatly accelerated the item search. In our case, since we were dealing with one-dimensional signals (hence significantly smaller than images), we were not especially seeking high speed execution. However in this approach, steps were considered as a key element to distinguish between medical staff and elderly activities. Therefore, by training a network to identify and characterize steps, the idea was to ensure that most of the information that was passed to the second sub-network of NurseNet was relevant. Moreover, as this step recognition part brought a separate training, it added a constraint to the overall model, hence reducing the chances of overfitting (see [75] for a discussion related to constraints and overfitting).

**Figure 6:**
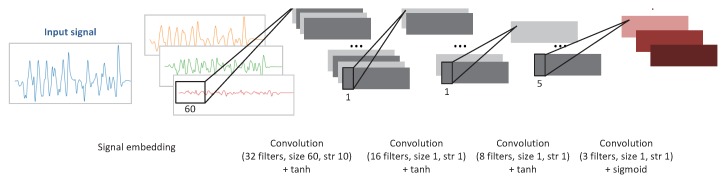
Architecture of the first part of the CNN, the SPN. Note that the last layer, which gives the output, is not present in the general NurseNet algorithm (Figure 2).

The architecture of the CNN used to train the first sub-network of NurseNet is presented in Figure 6. This CNN was made of:

- One 1D convolutional layer with 32 filters of size 60, with a stride of 10, followed by a BatchNorm layer, with the activation function hyperbolic tangent (tanh).
- One 1D convolutional layer with 16 filters of size 1, with a stride of 1, followed by a BatchNorm layer, with the activation function tanh.
- One 1D convolutional layer with 8 filters of size 1, with a stride of 1, followed by a BatchNorm layer, with the activation function tanh.
- One 1D convolutional layer with 3 filters of size 1, with a stride of 1, with the activation function sigmoid.

**Figure 7:**
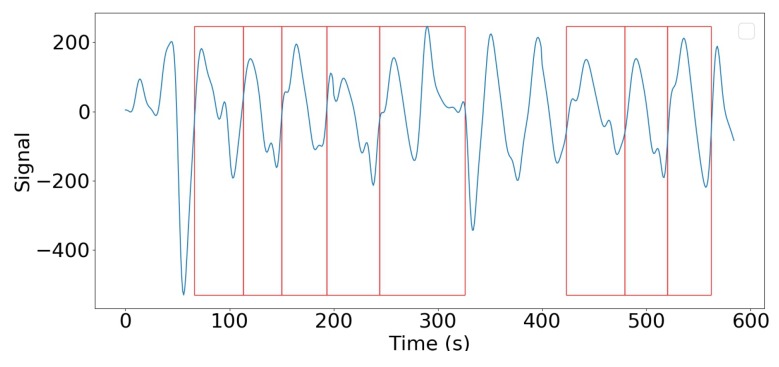
Example of the boxes’ delimiting steps in a walk signal. Only parts of the signal that can be undoubtedly connected to a step are labeled.

**Training set.** The training of this CNN was done as follows. For this task, we selected a subset of signals that only included easily-identifiable walks of the medical staff. Each of these signals was manually segmented by an expert using external sensor information, such as the video recordings of the equipped area. More precisely, boxes (denoted $b_{i}$) were manually labeled for each step of each embedded signal. Figure 7 gives an example of step delimiting boxes. The purpose of the SPN is to produce boxes $\hat{b}$ with a large Intersection over Union (IoU) score:

(5) $$\mathrm{IOU}\left(\hat{b}\right)≐\max_{j}\frac{|b_{j}\cap \hat{b}|}{|b_{j}\cup \hat{b}|}$$

i.e., boxes that are nearly equal to at least one of the ground-truth boxes. More precisely, the output of the network is a matrix $W\in R^{T\times K}$, with *T* being the signal length and *K* the number of different box sizes. Here, $W_{t}^{k}$ is interpreted as the probability that the box $b_{t}^{k}$ starting at time *t* and of size 20, 30, or 40 (for resp. k=1, 2, or 3) has a large IOU score. Those durations are chosen according to typical step durations of young healthy individuals. In line with [51], we define positive instances of boxes, i.e., $b_{t}^{k}$ such that $\mathrm{IOU}\left(b_{t}^{k}\right)>\sqrt{0.7}$, and negative instances, i.e., $b_{t}^{k}$ such that $\mathrm{IOU}\left(b_{t}^{k}\right)<\sqrt{0.3}$. It should be noted that in the original paper, authors used 0.7 (resp. 0.3) for the upper threshold (resp. the lower threshold). However, in their case, IOU was computed between 2D boxes, instead of 1D in our case. All other boxes were considered neutral boxes and did not participate in the training process. The loss function L is then defined as:

(6) $$L=\sum_{t}\sum_{k\in \left[1,2,3\right]}1_{\mathrm{IOU}\left(b_{t}^{k}\right)>\sqrt{0.7}}\log\left(W_{t}^{k}\right)+1_{\mathrm{IOU}\left(b_{t}^{k}\right)<\sqrt{0.3}}\log\left(1-W_{t}^{k}\right).$$

**Training process.** The weights of each layer were initialized using i.i.d. centered Gaussian variables with a standard deviation of 0.2. Regularization was done using dropout (with p=0.5) after each BatchNorm layer. Equation (6) was optimized using stochastic gradient descent with a learning rate of 10^−5^, decreasing geometrically (×0.9) every 10 epochs, and the Nesterov momentum strategy. The learning was stopped using the early stopping principle.

*Remark.* After its training, SPN achieved good, but lower than the state-of-the-art performance: for an IoU threshold of 0.7 (resp 0.9), it achieved an Average Precision (AP) of 85.2% (resp. 55.3%).

### 2.3. Data Collection

**Figure 8:**
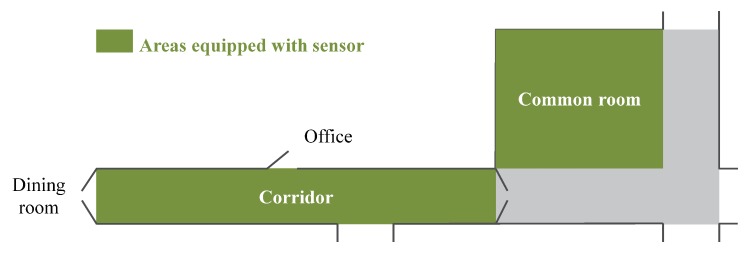
System installation in the nursing home.

The objective of NurseNet was to provide an unobtrusive tool to monitor physical activity of elderly individuals in a nursing home. To this end, a partner retirement house was chosen to collect the signals using the following setting. A corridor and a nearby common room were equipped with the system (Figure 8), and signals were recorded and labeled (event types and step boxes for the signals concerned) by an expert using two cameras. The areas, a corridor and a common room, were chosen for their accessibility (i.e., ease of installation of the system and event labeling) and the great frequency of people passing through. Indeed, the corridor is surrounded by the dining room, a caregiver office, and another corridor to another aisle of the nursing home, making it an important crossing point. Data were labeled according to the type of event (walking, rolling a cart, pushing a chair, etc.), the status of the person (patient or caregiver), and the number of persons on the floor.

**Figure 9:**
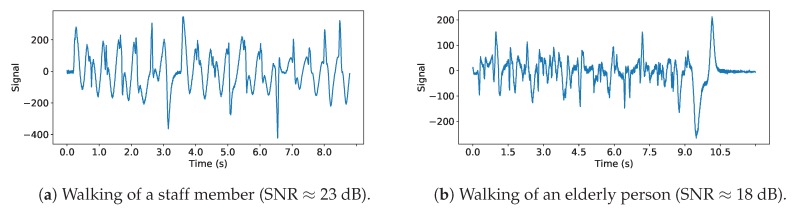
Example of signal of a young healthy person from the staff (**a**) and an elderly (**b**). The first observation is that the signal generated by the elderly footsteps has a significantly smaller amplitude, leading to a lower signal-to-noise ratio. We recognize some similarities between these signals as step patterns, as can be seen in (**b**). However as a whole, the (**b**) signal seems less repeatable than (**a**). This is probably due to the fact that the walking of an elderly can be less regular and less pronounced than a young healthy person whose feet leave and hit the floor at a regular pace.

The final database contained 93 labeled signals with a labeling resolution of 1 s for event labels (e.g walk, rolling a cart, etc.) and 10 ms for the steps of the concerned signals. Table 1 shows the number of events of each main class. It should be noted that the majority of signals are from caregivers. Consequently, it is crucial to identify them as accurately as possible in order not to alter a potential elderly activity monitoring system. Figure 9 shows two examples of signals recorded in the nursing home.

## 3. Results

In this section, we thoroughly evaluate the performance and behavior of NurseNet on multiple types of signals. First, the accuracy of NurseNet is studied over each subgroup of our database, and we take a closer look at misclassified signals. Then, a complete ablation analysis is done to show the relative improvement added by each part of the algorithm, and our approach is compared to a another robust off-the-shelf classification algorithm. The algorithm was tested and run with a Python implementation on a laptop with a 2.4-GHz Intel Core i7 processor. The training of the several stages (i.e., CDL, SPN, and NurseNet) took a few hours. However, the complete inference process was very fast (nearly instantaneous for a 10-s signal).

### 3.1. Performance Evaluation

The algorithm was evaluated by performing a stratified split of the dataset into training and testing sets (70 and 30%), resulting in similar distributions of data in both sets. The hyperparameters of CDL, SPN, and NurseNet were chosen using cross-validation on the training set. The separation was done using stratified random sampling along the three labels *single walk*, where one individual was walking on the sensor, *multiple walks*, where strictly more than one individual was walking at the same time on the sensor, and *others*, where one non-walk-related event occurred (e.g., pushing a wheelchair or a cart). The signals were labelled as *staff* if all the persons involved were parts of the medical staff and *elderly* otherwise. The demographics are detailed in Table 2.

**Figure 10:**
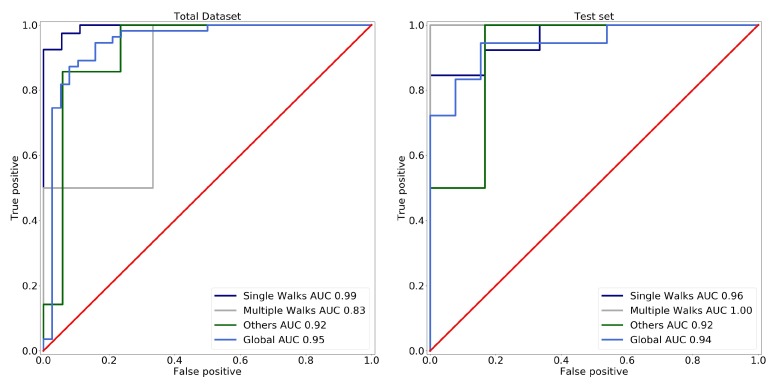
ROC obtained when classifying between medical staff and elderly individuals for different activities (single individual walk, multiple individuals walks, other) using NurseNet on (**left**) the entire dataset and (**right**) the test set. Performances are similar in both cases.

**Figure 11:**
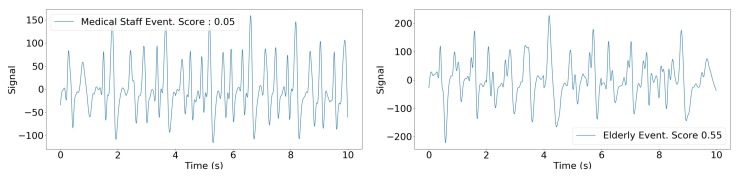
Examples of signals misclassified by NurseNet. (**Left**) Nurse pushing a wheelchair. Note that the signal is irregular. (**Right**) Elderly walking. Contrary to the previous example (Figure 9), this signal presents higher periodicity and amplitude, and the recorded individual is healthier.

NurseNet achieved a global AUC of 0.94(±0.02) on the test set for the classification between staff and non-staff activities. This high performance was evenly distributed on all the labels, as shown by Figure 10. Additionally, it should be noted that the accuracy of NurseNet was similar for both the test set and the entire dataset, which highlights the generalizability of our approach. Table 3 presents two examples of classification obtained by NurseNet for different thresholds, and Figure 11 shows examples of misclassified signals.

### 3.2. Ablation Analysis

**Figure 12:**
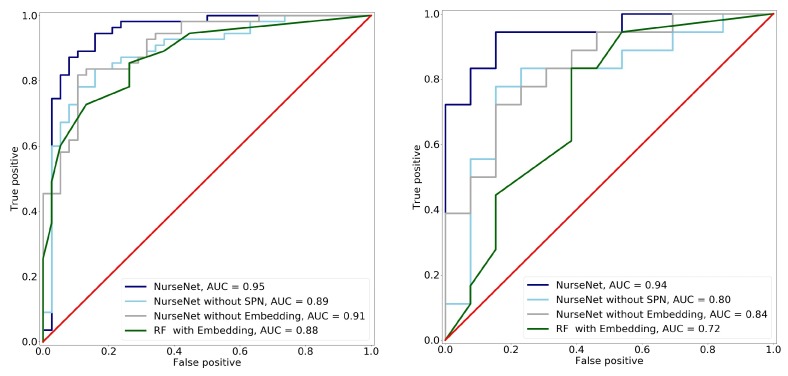
ROC obtained when classifying staff/elderly activities using the different variants of NurseNet and RF on (**left**) the entire dataset and (**right**) the test set. The corresponding AUCs are displayed in the figure legend. Note that NurseNet outperformed all the other algorithms in both cases. It also generalized well, as it had consistent performances between the test set and the entire dataset, contrary to the others.

This subsection aims to perform an ablation analysis on NurseNet and compare it to non-deep learning-based approaches. To achieve this result, a version of NurseNet was trained without the SPN pre-training part. In other words, the network was trained from scratch on the final classification task. Another version of NurseNet was trained, in which there was neither dictionary embedding nor SPN pre-training, i.e., it used directly the raw signal as an input, and the whole CNN was trained on the classification task. Finally, NurseNet was compared to a Random Forest (RF) algorithm, which is known to provide good results. The RF features were 10-s padded signals decomposed using dictionary embedding and the atoms learned in Section 2.2.2. Results are presented in Figure 12.

It is important to notice that while all the previously-described algorithms achieved good performance on the entire dataset (which includes the training set), their AUCs significantly dropped on the test set, hence suggesting significant overfitting. This was particularly true for RF, whose AUC was only 0.72(±0.04) on the test set, highlighting the advantage of a CNN-based approach in our setting. Additionally, NurseNet significantly outperformed both its simplified versions, which illustrates the advantages of our training method. Finally, it should be noted that NurseNet without embedding, which is a CNN directly applied to the raw signal, achieved the surprisingly high AUC of 0.84(±0.03).

## 4. Discussion

**Classification across all labels.** It is interesting to note that NurseNet performed well across all labels described in Table 2. This might seem counter-intuitive at first as the algorithm was pretrained toward detecting steps and some signals contained non-gait-related activities (e.g., pushing a cart). A possible explanation comes from the fact that all signals contained some elements of walks. In the example where a nurse was pushing a cart, while not obvious to the untrained eye, it was possible that some characteristics of the medical staff walk were encoded in the signal. Therefore, the features learned by the SPN could be relevant to correctly assess the signals.

**Multiple training.** Interestingly, the CNN without the dictionary encoding, nor SPN pre-trainings performed significantly better than the CNN with the sole dictionary encoding. This may be explained as follows. Both the dictionary encoding and the SPN are two complementary stages of step detection: the dictionary extracted features linked to step characteristics, and the SPN was trained to use these features to segment the signal into steps. On the one hand, when the CNN was used with only dictionary embedding, the training task became more complex as the network had to learn how to use these features to recognize steps while trying to classify these signals. On the other hand, the regular CNN algorithm could use any non-step-related features to perform the classification. Consequently, its objective function was significantly less constrained and had a larger number of reasonable local minima, making it easier to train. However, it should be noted that when properly guided toward a step-related solution (NurseNet), the CNN appeared to perform even better, which emphasized the interest of our approach.

**NurseNet****vs. shallow.** On this classification task, the random forest model gave reasonable performance on the test set (AUC 0.72). However, it was significantly lower than its overall performance (AUC 0.88), which indicates that it was overfitting. We observed similar behavior for most off-the-shelf algorithms (e.g., SVM). This may be explained by the complex nature of the signal: while NurseNet (as well as other deep network algorithms) can learn relevant features to describe the signal, shallow algorithms tackle a significantly harder task. It is the authors belief that with properly-designed features, RF may achieve performance similar to CNN. However, due to the very good AUC obtained by NurseNet, we chose not to push further into the manual feature engineering, as it is always possible to use features learned from NurseNet to improve the results of other algorithms.

**In-depth classification.** As seen in Section 3, NurseNet was able to separate medical staff and elderly activities with very high accuracy (AUC 0.94). Future work will focus on separating different categories of activity as each one of them entails different indicators regarding elderly activity monitoring. However, this task requires a significantly larger dataset, and therefore, the installation of NurseNet on multiple locations.

**Figure 13:**
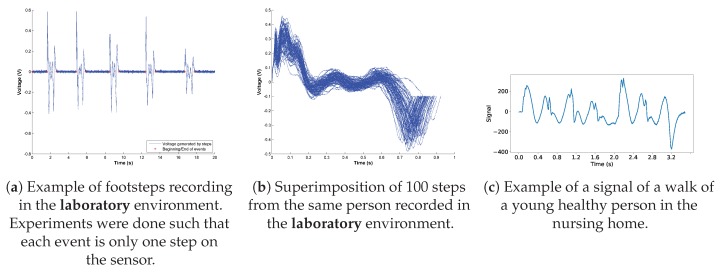
When considering signals recorded in laboratory conditions (**a**,**b**), we notice a certain regularity and good repeatability of events that are not of the same quality when looking at signals taken in the nursing home (**c**). Figure (**a**,**b**) were taken with the author’s agreement.

**Signal Quality.** Serra et al. [10] used a similar sensor to NurseNet and showed examples of footstep recordings that proved to be very reliable in their interpretability. For instance, different phases of a footstep could be seen: heel, toe, weight transfer, and foot removal. Moreover, these footsteps were recorded multiple times with different persons, showing that the generated signal was repeatable, illustrating it with a superimposition of several footsteps of one person (see Figure 13). However, in our application, the output was of significantly lower quality. There were multiple factors that may account for this difference. The authors of [10] recorded their signals in a controlled environment: a single band was directly linked to an acquisition unit dedicated to laboratory prototyping. These laboratory conditions lead to high-quality output signals, and events such as walks could be easily decomposed. However, in our setting and due to scale and cost issues, the signal was collected on multiple large surfaces and sent through a processing unit designed to perform multiple tasks including the amplification of the signal, which may alter the quality of the data. Moreover, in addition to the previously-acknowledged limitations (i.e., non-homogeneousness piezoelectric coefficient across space), the system appeared to be sensitive to humidity. Indeed, in this setting, the signal was collected through crimps hooked to the sensor and linked to a printed circuit. This connection was vulnerable to humidity that may be in the surfaces in contact with the system (e.g., adjacent walls), which may even lead to corrosion. Besides, this humidity may enter in contact with the edges of the sensor, thus creating an additional resistance between the two sides of the device. Depending on the characteristics of the humidity, these two phenomenons may significantly alter the output of the unit. Future improvement of the sensor would aim to reduce these effects.

**Individual identification.** While NurseNet achieved very high accuracy for classifying the elderly and medical staff, it is not able at the moment to recognize individuals. This is due to the fact that the dataset used only included a handful of signals for each individual. However, it is the authors’ belief that with significantly more data, NurseNet can be improved to distinguish between individuals, albeit at a lower accuracy than the staff/elderly separation. Future work will aim at improving NurseNet in that regard. It should also be noted that despite this limitation, it was already possible to assign the activity to a person with reasonable accuracy using external information, using for instance the location of the system (e.g., if the system is installed near or inside a person’s room).

## Figures and Tables

**Table 1:** Number of instances in the dataset. Signals have an average duration of 10 s. Note that in the *walking, >1 person* category, all the walkers were either all elderly or all medical staff.

Event	Number of Instances
Walking, 1 person, staff	42
Walking, 1 person, elderly	16
Walking, >1 person	11
Wheelchair	9
Wheelchair pushed by another	5
Walking with a cart	5
Other events	5

**Table 2:** Quantity and nature of the signals contained in our dataset. Data were split into training and test using stratified random sampling along the classes single walk, multiple walks and other.

Label	Staff	Elderly
Single Walk	40	18
Multiple Walks	8	3
Other	7	17

**Table 3:** Confusion matrix for different thresholds τ, i.e., activity is classified as staff if the network produces an output greater than or equal to τ. Note that the staff activities that are confused by the networks are essentially the nurse pushing medical material such as wheelchairs or carts on the sensor.

		Staff			Elderly		
		Single Walk	Multiple Walks	Other	Single Walk	Multiple Walks	Other
	τ=0.2						
Test	Classified Staff	13	3	1	2	0	0
	Classified Elderly	0	0	1	4	1	6
All	Classified Staff	40	7	5	4	1	1
	Classified Elderly	0	1	2	14	2	16
	τ=0.4						
Test	Classified Staff	12	2	1	1	0	0
	Classified Elderly	1	1	1	5	1	6
All	Classified Staff	39	3	3	1	0	1
	Classified Elderly	1	5	4	17	3	16

## Abbreviations

The following abbreviations are used in this manuscript:

NurseNet	Non-invasive Unit Recognition System for the Elderly Network
SPN	Step Proposal Network
RPN	Region Proposal Network
CNN	Convolutional Neural Network
CDL	Convolutional Dictionary Learning
RF	Random Forest
